# The CT-guided percutaneous drainage of pancreatic pseudocyst accompanied by pseudoaneurysm: A case report

**DOI:** 10.1097/MD.0000000000037402

**Published:** 2024-03-08

**Authors:** Qimin Yang, Bing Li, Bai Jin Tao Sun, Xiangkai Zhong, Zhiqiang Qiu, Hanfeng Yang

**Affiliations:** a Department of Radiology, Affiliated Hospital of North Sichuan Medical College, 1 Maoyuan south Road, Nanchong, Sichuan, People’s Republic of China.

**Keywords:** CT-guided percutaneous drainage, pancreatic pseudocyst, pseudoaneurysm

## Abstract

**Rationale:**

This case report discusses the CT-guided percutaneous drainage of a pancreatic pseudocyst accompanied by a pseudoaneurysm. Pancreatic pseudocysts can erode the peripancreatic artery and produce pseudoaneurysms. This is rare, but it can be life-threatening.

**Patient concerns:**

The case presented involves a 58-year-old female who was diagnosed with pancreatic cancer and underwent surgical treatment. She presented with hematochezia, dizziness, and hypodynamic findings with no obvious cause. Imaging revealed a pancreatic pseudocyst and small arterial aneurysms. To reduce the risk of aneurysm rupture, the patient underwent transcatheter arterial coil embolization. Three days later, CT-guided catheter drainage was performed to reduce the erosion of the arterial wall caused by pancreatic fluid.

**Diagnoses:**

The contrast-enhanced-CT imaging showed a round, slightly high-density lesion in the cyst, suggesting the presence of a pseudoaneurysm.

**Interventions:**

The patient was sent for another transcatheter arterial embolization with coils and n-butyl-2-cyanoacrylate.

**Outcomes:**

After receiving the transcatheter arterial embolization, the patient had no serious bleeding or other complications.

**Lessons:**

Early detection and accurate assessment of pseudoaneurysms are essential for appropriate management. This case shows that contrast-enhanced CT is necessary before CT-guided percutaneous drainage of pancreatic pseudocysts. It also shows that, due to the many complications that pancreatic pseudocysts may cause, appropriate treatment of pseudocysts complicated with pseudoaneurysm has important clinical significance.

## 1. Introduction

A pancreatic pseudocyst, a collection of fluids in the peripancreatic tissue, is rich in pancreatic enzymes and contains no solid elements. It is always surrounded by a well-defined wall consisting of granulated and fibrous tissue. Diagnosis can always be made based on its morphologic criteria.^[1]^ The incidence of pancreatic pseudocyst is extremely low, at approximately 1.6% to 4.5% per 100,000 adults per year.^[2]^ Arterial pseudoaneurysms within pancreatic pseudocysts are rare, but may become life-threatening when the pseudoaneurysm develops intracystic hemorrhage. In these cases, mortality can reach 40%.^[3,4]^ Pseudoaneurysms are formed due to the inflammation of the pancreas and the tissue surrounding it, pseudocyst formation, the exosmosis of proteolytic enzymes, and extensive necrosis. A pancreatic pseudocyst can erode its way into the peripancreatic artery, which transforms the pseudocyst into a large pseudoaneurysm.^[3,5,6]^ CT-guided percutaneous drainage of pancreatic pseudocysts was first attempted late in the last century, and it allowed successful external drainage.^[7]^ Using this method, the puncture can be made under real-time CT image guidance, which makes the procedure safer and more efficient.

In what follows, we present the case of a 58-year-old female with a common hepatic aneurysm within a pancreatic pseudocyst 3 days after embolization of the common hepatic aneurysm. A pseudoaneurysm was found during percutaneous drainage of the pancreatic pseudocyst under CT guidance.

## 2. Case report

This case report was approved by the Affiliated Hospital of North Sichuan Medical College ethics committee. A 58-year-old female was diagnosed with pancreatic cancer in our hospital in January 2021. She underwent surgical treatment 4 months later. After the operation, the patient underwent immunotherapy. Three months after the surgery, the patient experienced abdominal pain and vomiting without bloody stool, dizziness, fatigue, or other manifestations.

After the patient was admitted to the hospital, upper abdominal enhanced-MRI revealed a pancreatic pseudocyst at the pancreatic tail and several small arterial aneurysms at the far end of the common hepatic artery. Her blood pressure was 122/73 mm Hg and she had a regular heartbeat (78 bpm). The patient was 150 cm tall and weighed 39 kg (BMI 19.1). The results of the laboratory tests were as follows: reduced red blood cell count (2.93 × 10¹²/L), hemoglobin (81 g/L), hematocrit (0.225); AST (46 U/L), ALT (64 U/L), albumin (36.7 g/L), and CA19-9 (156 U/ml).

**Figure 1.**
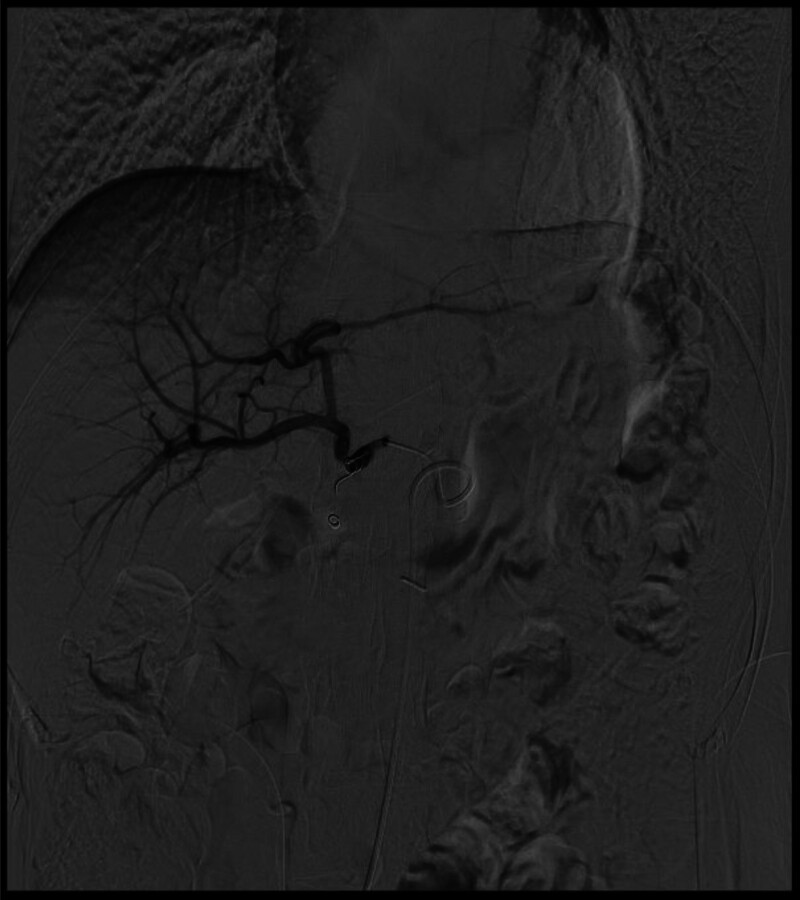
Digital subtraction angiogram showing no aneurysm after transcatheter arterial embolization of common hepatic artery.

**Figure 2.**
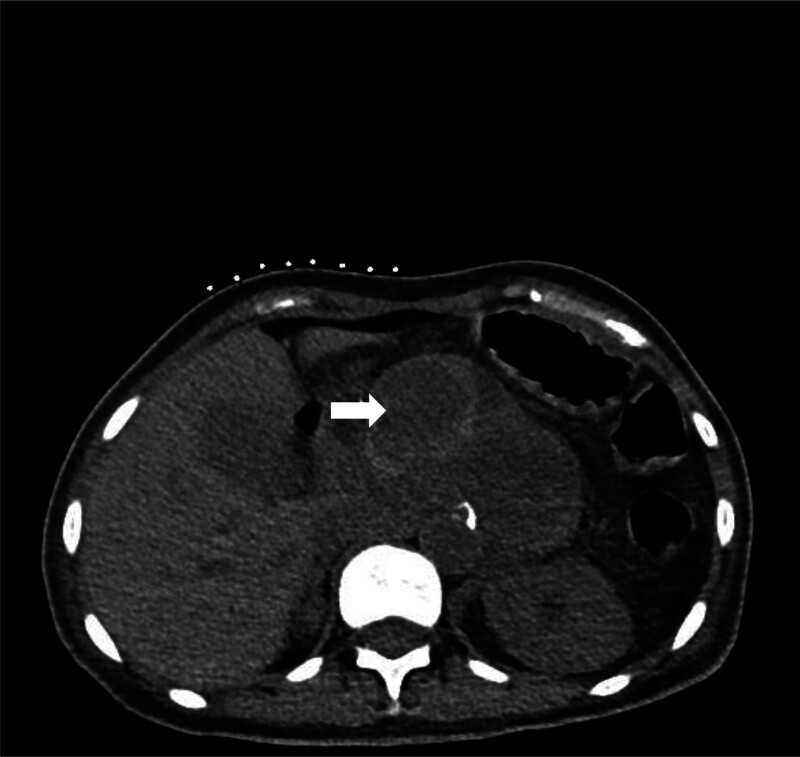
Unenhanced CT scan before the procedure showing a round, slightly high-density lesion (arrow).

**Figure 3.**
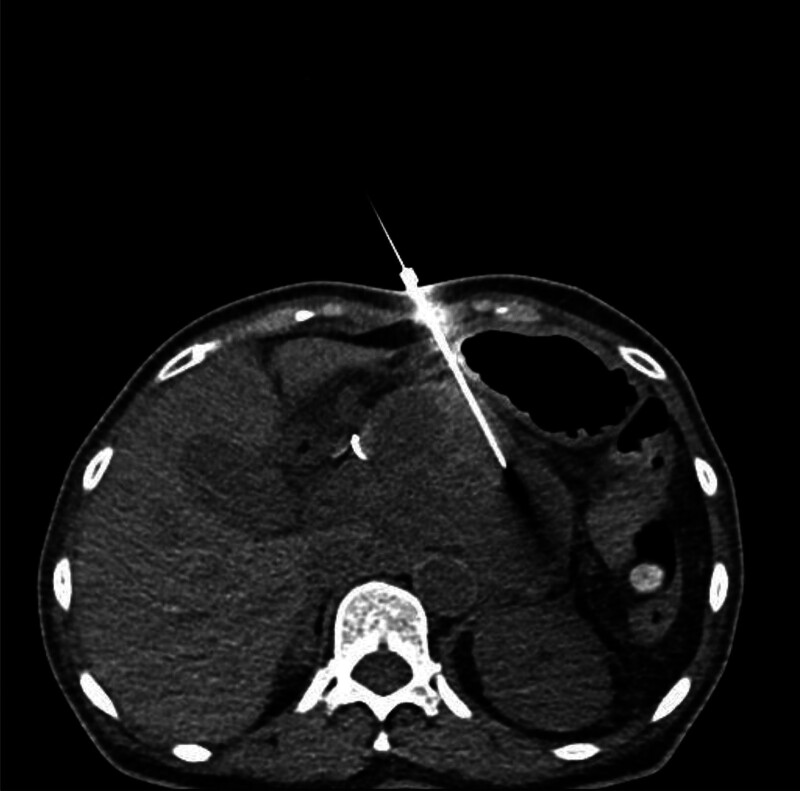
A 19-gauge needle guided by real-time CT imaging, with a puncture path from the outside of the lesion into the pseudocyst.

**Figure 4.**
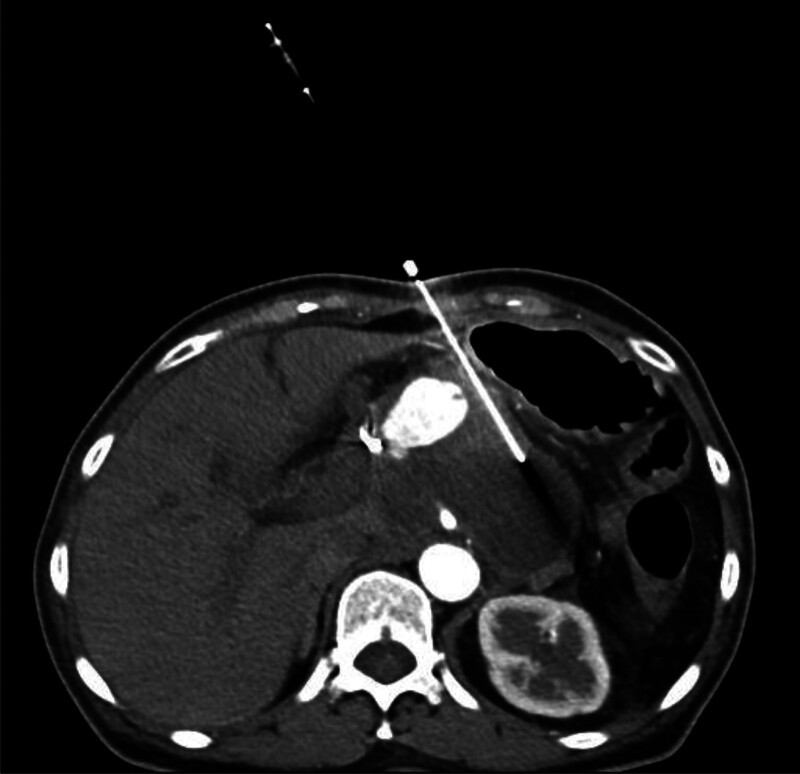
Contrast-enhanced CT scan of the abdomen during the procedure, showing a circular lesion with obvious enhancement.

**Figure 5.**
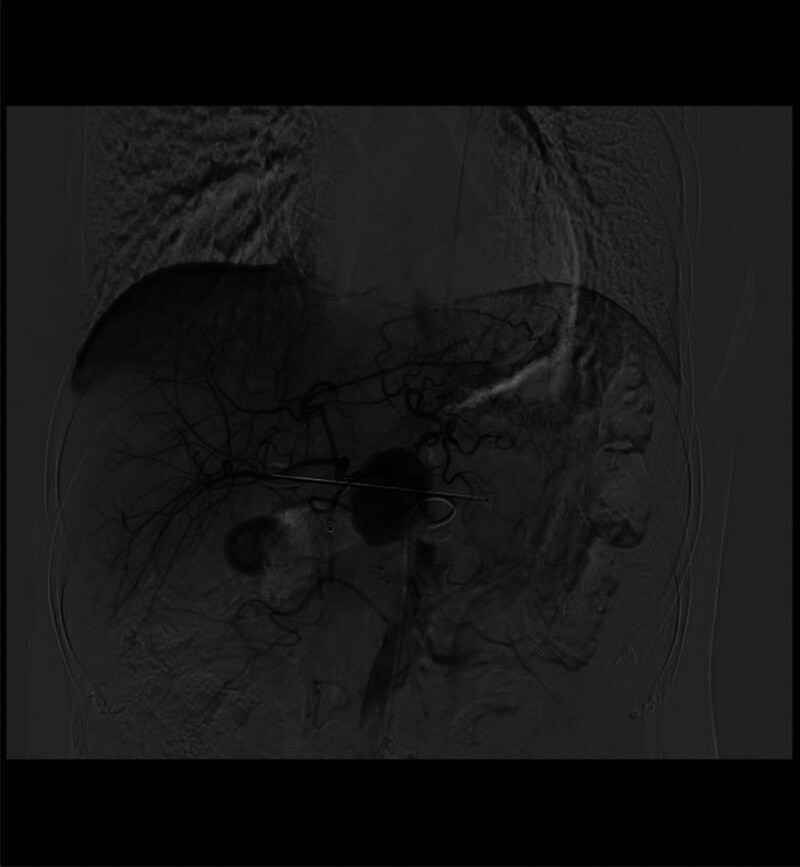
Digital subtraction angiogram showing the pseudoaneurysm during the emergency transcatheter arterial embolization.

**Figure 6.**
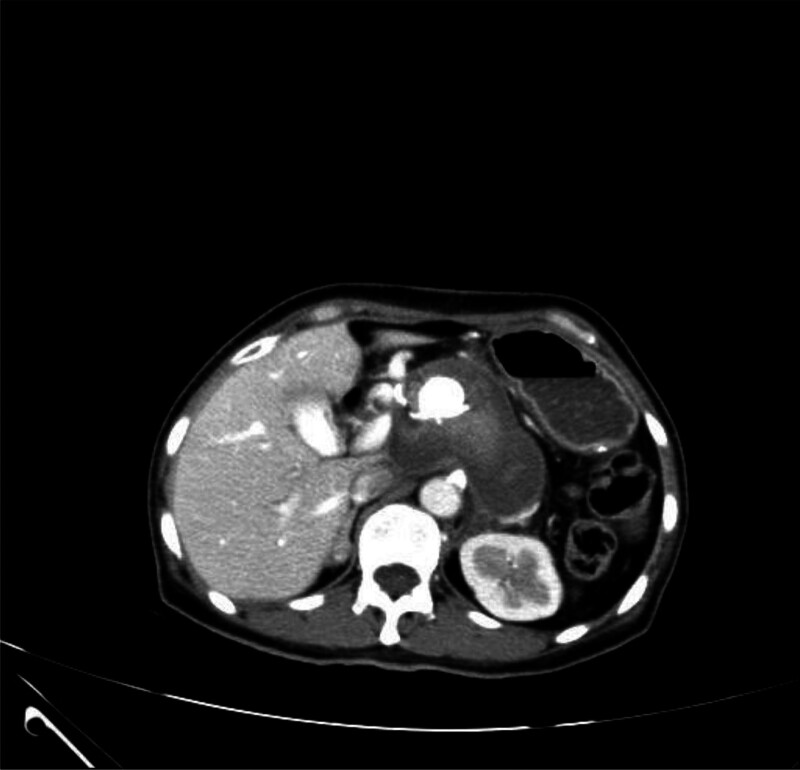
Postoperative CT showing no hemorrhage.

In order to reduce the risk of aneurysm rupture and bleeding, the patient underwent transcatheter arterial coil embolization. After embolization, no aneurysms were found using digital subtraction angiography (DSA) (Fig. 1). Three days after arterial embolization, in order to reduce the erosion of arterial wall caused by pancreatic fluid in the pancreatic pseudocyst, clinicians recommend CT-guided catheter drainage treatment of the pancreatic pseudocysts. The patient lay on the CT table in the supine position. An unenhanced CT scan was performed for positioning, and a round, slightly high-density lesion (arrow) could be seen in the cyst (Fig. 2). Because the patient had just undergone and embolization, the interventional radiologist thought that the lesion was a postoperative change. However, to be on the safe side, the puncture path was set outside the lesion and then directed into the pseudocyst (Fig. 3). After the needle entered the cyst, bloody liquid was ejected from the puncture point at high pressure after the needle core was withdrawn. The needle core was replaced immediately and a contrast-enhanced CT scan was performed, showing obvious enhancement at a readily visible circular lesion. This was considered a pseudoaneurysm (Fig. 4). The patient was immediately sent to the DSA room for emergency pseudoaneurysm embolization with coils and n-Butyl-2-cyanoacrylate (Fig. 5). No pseudoaneurysm was found after the operation (Fig. 5). Then, the puncture needle path was sealed with gelfoam and the needle was withdrawn. No signs of bleeding were observed in the postoperative CT scan (Fig. 6). The patient successfully recovered from a life-threatening situation. She was discharged home without any complications after 1 week. And was followed up for more than 2 years and no long-term effects were observed.

## 3. Discussion

In this case report, we discuss a rare case of pseudoaneurysm secondary to the embolization of aneurysms in a pancreatic pseudocyst. Pancreatic pseudocysts are common in both acute and chronic pancreatitis. The incidence of pancreatic pseudocyst among patients with chronic pancreatitis is 20% to 40%.^[8]^ Pancreatic pseudocysts are collections of enzyme-rich fluid in the peripancreatic tissue.^[1]^ Pancreatic pseudocysts can cause secondary complications such as infection, stenosis of the common bile duct, pseudoaneurysm-induced hemorrhage, and obstruction of the stomach.^[9]^ Arterial pseudoaneurysm within a pancreatic pseudocyst is rare and life-threatening, and is often caused when the pancreatic fluid in pseudocysts erodes the artery wall. The splenic artery is most commonly involved (in approximately 30%–50% of cases).^[3,5,6,10]^

Pancreatic pseudocysts usually require no treatment; most of them regress spontaneously. Some, especially those larger than 6 cm, require treatment to prevent complications such as infection, rupture, and hemorrhage.^[10–12]^ Endoscopic ultrasound-guided drainage is considered a suitable drainage approach for uncomplicated pseudocysts. However, image-guided drainage might be more suitable for complicated cases. Image-guided percutaneous drainage (usually performed under CT or ultrasound control) is a reliable and relatively inexpensive drainage method that involves either percutaneous aspiration or percutaneous catheter drainage.^[4,10,13]^

In this case, the patient underwent transcatheter arterial embolization therapy to resolve the arterial aneurysm at the far end of common hepatic artery. We then performed CT-guided percutaneous drainage of pancreatic pseudocyst. Recent reports have suggested that the risk of rupture in aneurysms in the gastroduodenal aneurysms and hepatic artery (HAA) is greater than previously believed, and they require proactive therapy regardless of the size of the aneurysm.^[14]^ Endovascular therapy is considered the first choice for treating HAA, and coil embolization is recommended for patients with intrahepatic aneurysm.^[15]^ This patient only underwent coil embolization at first, and the results may not have been very successful. Coil embolization may be the most common technique, but it is hard to fully fill the vessels, and sometimes coil embolization cannot reach the smaller arteriole branches.^[14,16]^

In this case, the fluid in the pancreatic pseudocyst, which is rich in pancreatic enzymes, leaked and eroded into peripancreatic artery, producing a new pseudoaneurysm. This situation was complicated. During the second round of transcatheter arterial embolization therapy, we used coil embolization and the injection of liquid embolic agents to resolve it. Embolization using liquid embolic agents is an effective method, especially in complicated cases. It fills the vessels to a greater extent than coil embolization can. However, the cytotoxicity generated by organic solvents can cause severe complications, including death.^[17–19]^ Covered stents might produce better results in clinical settings.^[16,20]^

The unenhanced CT scan performed before catheter drainage showed a round, slightly high-density lesion in the cyst. This did not initially worry us because the patient had undergone embolization only 3 days earlier. This is regarded as a postoperative change rather than an infrequent pseudoaneurysm. To be safe, we set the puncture path the outside of the lesion into the pseudocyst. Our case suggests that a contrast-enhanced CT scan is necessary, especially under abnormal conditions. Contrast-enhanced CT and DSA have been widely used to detect pseudoaneurysms. Unenhanced CT scans may show a round, low-density structure arising from the parent artery, and high-density lesions inside or around the pseudoaneurysm may suggest hemorrhage.^[21]^ Contrast-enhanced CT may display a cyst filled with contrast material, which may increase in intensity rapidly as the contrast agent is injected. This cyst may have a density similar to those of the aorta and other larger splanchnic arteries.^[21,22]^ We must assess the imaging manifestations of pseudoaneurysm in order to produce accurate judgment for future treatments.

When we found the pseudoaneurysm, we did not withdraw the puncture needle. This is crucial, especially in the short term, as it prevents extensive hemorrhage resulting from the rupture of the pseudoaneurysm. This allows for a window of time to perform subsequent therapeutic interventions. We sent the patient to the DSA operating room for embolization treatment immediately. After confirming the success of embolization, the puncture needle was withdrawn. The patient had no serious bleeding or other complications.

## 4. Conclusion

Arterial pseudoaneurysm within a pancreatic pseudocyst is rare and may involve high mortality rates when hemorrhage occurs. Correct diagnosis and appropriate treatment are of considerable clinical importance.

## Acknowledgments

We thank LetPub (www.letpub.com) for its linguistic assistance during the preparation of this manuscript.

## Author contributions

**Writing—original draft:** Qimin Yang

**Writing—review & editing:** Hanfeng Yang

**Investigation:** Bing Li, Bai Jin Tao Sun, Xiangkai Zhong, Zhiqiang Qiu

## References

[1] Banks PA Bollen TL Dervenis C. Classification of acute pancreatitis--2012: revision of the Atlanta classification and definitions by international consensus. Gut. 2013;62:102–11. 23100216 10.1136/gutjnl-2012-302779

[2] Sandy JT Taylor RH Christensen RM. Pancreatic pseudocyst. Changing concepts in management. Am J Surg. 1981;141:574–6. 7223950 10.1016/0002-9610(81)90052-0

[3] Hsu J Yeh C-N Hung C-F. Management and outcome of bleeding pseudoaneurysm associated with chronic pancreatitis. BMC Gastroenterol. 2006;6:3. 16405731 10.1186/1471-230X-6-3 PMC1361773

[4] Phillip V Braren R Lukas N. Arterial pseudoaneurysm within a pancreatic pseudocyst. Case Rep Gastroenterol. 2018;12:513–8. 30283285 10.1159/000492459 PMC6167644

[5] Flati G Andrén-Sandberg A La Pinta M. Potentially fatal bleeding in acute pancreatitis: pathophysiology, prevention, and treatment. Pancreas. 2003;26:8–14. 12499910 10.1097/00006676-200301000-00002

[6] Flati G Salvatori F Porowska B. Severe hemorrhagic complications in pancreatitis. Ann Ital Chir. 1995;66:233–7. 7668500

[7] Lee MJ Wittich GR Mueller PR. Percutaneous intervention in acute pancreatitis. Radiographics. 1998;18:711–24; discussion 728. 9599393 10.1148/radiographics.18.3.9599393

[8] Baillie J. Pancreatic pseudocysts (part I). Gastrointest Endosc. 2004;59:873–9. 15173808 10.1016/s0016-5107(04)00354-2

[9] Andrén-Sandberg A Dervenis C. Pancreatic pseudocysts in the 21st century. Part II: natural history. JOP. 2004;5:64–70. 15007187

[10] Zerem E Hauser G Loga-Zec S. Minimally invasive treatment of pancreatic pseudocysts. World J Gastroenterol. 2015;21:6850–60. 26078561 10.3748/wjg.v21.i22.6850 PMC4462725

[11] Zerem E Imamović G Omerović S. Percutaneous treatment for symptomatic pancreatic pseudocysts: long-term results in a single center. Eur J Int Med. 2010;21:393–7. 10.1016/j.ejim.2010.06.015 20816592

[12] Gumaste VV Aron J. Pseudocyst management: endoscopic drainage and other emerging techniques. J Clin Gastroenterol. 2010;44:326–31. 20142757 10.1097/MCG.0b013e3181cd9d2f

[13] Yasuda I Iwata K Mukai T. EUS-guided pancreatic pseudocyst drainage. Dig Endosc. 2009;21(Suppl 1):S82–6. 19691744 10.1111/j.1443-1661.2009.00875.x

[14] Obara H Kentaro M Inoue M. Current management strategies for visceral artery aneurysms: an overview. Surg Today. 2020;50:38–49. 31620866 10.1007/s00595-019-01898-3 PMC6949316

[15] Chaer RA Abularrage CJ Coleman DM. The Society for Vascular Surgery clinical practice guidelines on the management of visceral aneurysms. J Vasc Surg. 2020;72:3S–39S. 32201007 10.1016/j.jvs.2020.01.039

[16] Fan L Duan M Xie Z. Injectable and radiopaque liquid metal/calcium alginate hydrogels for endovascular embolization and tumor embolotherapy. Small. 2020;16:e1903421. 31762193 10.1002/smll.201903421

[17] Dudeck O Jordan O Hoffmann KT. Organic solvents as vehicles for precipitating liquid embolics: a comparative angiotoxicity study with superselective injections of swine rete mirabile. AJNR Am J Neuroradiol. 2006;27:1900–6. 17032862 PMC7977874

[18] Mottu F Laurent A Rufenacht DA. Organic solvents for pharmaceutical parenterals and embolic liquids: a review of toxicity data. PDA J Pharm Sci Technol. 2000;54:456–69. 11107838

[19] Zhou X Li Y Chen S. Dynamic agent of an injectable and self-healing drug-loaded hydrogel for embolization therapy. Colloids Surf B Biointerfaces. 2018;172:601–7. 30219579 10.1016/j.colsurfb.2018.09.016

[20] Hu J Altun I Zhang Z. Bioactive-tissue-derived nanocomposite hydrogel for permanent arterial embolization and enhanced vascular healing. Adv Mater. 2020;32:e2002611. 32578337 10.1002/adma.202002611 PMC7491606

[21] Saad NEA Saad WEA Davies MG. Pseudoaneurysms and the role of minimally invasive techniques in their management. Radiographics. 2005;25(Suppl 1):S173–89. 16227490 10.1148/rg.25si055503

[22] Kim HC Yang DM Kim HJ. Computed tomography appearances of various complications associated with pancreatic pseudocysts. Acta Radiol. 2008;49:727–34. 19143058 10.1080/02841850802104932

